# Critical Care Nutrition from a Metabolic Point of View: A Narrative Review

**DOI:** 10.3390/nu17081352

**Published:** 2025-04-15

**Authors:** Takehiko Oami, Akiyuki Yamamoto, Shigenobu Ishida, Kengo Kondo, Nanami Hata, Taku Oshima

**Affiliations:** 1 Department of Emergency and Critical Care Medicine, Chiba University Graduate School of Medicine, Chiba 260-8677, Japan; seveneleven711thanks39@msn.com (T.O.);; 2 Institute for Advanced Academic Research, Chiba University, Chiba 263-8522, Japan; 3 Research Institute of Disaster Medicine, Chiba University, Chiba 263-8522, Japan

**Keywords:** critical illness, energy expenditure, microbiota, metabolomics

## Abstract

**Background:** Critical illness induces profound metabolic alterations, characterized by a hypermetabolic state, insulin resistance, protein catabolism, and gut barrier dysfunction, which contribute to increased morbidity and mortality. Emerging evidence highlights the role of the gut microbiome and its metabolites in modulating systemic inflammation and immune responses during critical illness. This narrative review explores the metabolic evolution of critically ill patients, the impact of gut dysbiosis on disease progression, and the potential role of nutrition in modulating metabolism and improving patient outcomes. **Methods:** A comprehensive literature search was conducted across PubMed and Google Scholar for articles published up to February 2025. Search terms included “critical illness”, “metabolism”, “gut microbiota”, “nutrition”, and related keywords. Articles published in English addressing metabolic alterations, microbiome changes, and nutritional strategies in critically ill patients were included. After screening for eligibility, relevant articles were synthesized to outline current knowledge and identify gaps. **Results:** Metabolic changes in critical illness progress through distinct phases, from catabolism-driven hypermetabolism to gradual recovery. Gut dysbiosis, characterized by a loss of microbial diversity and increased gut permeability, contributes to systemic inflammation and organ dysfunction. Nutritional strategies, including enteral nutrition, probiotics, prebiotics, and metabolomics-driven interventions, may help restore microbial balance, preserve gut barrier integrity, and modulate immune and metabolic responses. Future nutrition therapy should focus on metabolic modulation rather than solely addressing nutrient deficits. **Conclusions:** Advances in gut microbiome research and metabolomics offer new avenues for personalized nutrition strategies tailored to the metabolic demands of critically ill patients. Integrating these approaches may improve clinical and functional recovery while mitigating the long-term consequences of critical illness.

## 1. Introduction

Critical illness is generally characterized by systemic inflammation, due to acute pathologies such as sepsis, severe burns, trauma, severe cardiovascular diseases, and acute organ failures [1]. Survival is now readily accomplished by advanced patient care and treatment, but usually in exchange for severe physical and psychological disabilities [2]. This phenomenon, known as intensive care unit (ICU)-acquired weakness, is associated with longer hospital stays and mortality. Metabolic alterations play an important role in the survival process, by utilizing endogenous and exogenous energy sources efficiently during the different phases of the acute systemic inflammation [3,4]. Nutrition therapy is suggested as a tool to ameliorate these consequences, mainly by providing sufficient energy, protein, and other vital nutrients for sustaining the inflammatory response and preserving the bodily structures [3]. However, the metabolic alterations that occur during critical illness are complex and dynamic, involving hypermetabolism, insulin resistance, and protein catabolism, complicating the development of effective nutritional strategies.

Recent studies have highlighted the critical roles of the gut, influencing both immune and metabolic homeostasis [5,6]. The gut microbiota, comprising trillions of microorganisms, plays an essential role in nutrient absorption, the regulation of energy metabolism, and immunomodulation through the production of gut-derived metabolites such as short-chain fatty acids (SCFAs) [7]. In critical illness, gut dysbiosis, characterized by a reduction in microbial diversity and disruption of the gut barrier, contributes to systemic inflammation, impaired immune function, and organ dysfunction [8,9,10]. Nutrition, especially when provided through the enteral route, can also act as a modulator of these metabolic processes by affecting both the microbiota composition and gut barrier structures [5,11].

Despite the growing recognition of the interconnection between the altered gut microenvironment, nutrient metabolism, and clinical outcomes, most existing reviews have focused on the nutritional needs of critically ill patients rather than exploring the underlying metabolic mechanisms and evaluating how targeted nutritional strategies can modulate metabolic alterations [12,13,14]. These reviews have often overlooked the complex interplay between metabolic changes, gut microbiota, and nutrition in critical care. Moreover, few reviews have integrated evidence from recent advances in omics technologies, including microbiome analyses and metabolomics, which offer novel perspectives on host–microbe interactions and nutrient utilization in critical illness [15,16,17].

Therefore, this narrative review aims to synthesize evidence from basic research, clinical observational studies, and randomized controlled trials to provide a comprehensive understanding of the metabolic evolution in critical illness and the role of nutrition in modulating these metabolic changes based on the altered gut microenvironment. The review will also highlight potential avenues for personalized nutritional strategies based on advances in gut microbiome and metabolomics research. By addressing these knowledge gaps, this review seeks to offer new insights into optimizing nutrition therapy to improve both short- and long-term outcomes in critically ill patients.

## 2. Methods

This narrative review includes basic research, clinical observational studies, and randomized controlled trials relevant to the review topics. The literature search was conducted using PubMed and Google Scholar, limited to English-language publications without a restriction on publication date. The search terms included “critical illness”, “metabolism”, “gut microbiota”, “nutrition”, and related keywords. Non-English publications, conference abstracts, and studies unrelated to metabolic or microbiome-aspects papers were excluded After retrieving full-text articles, we reviewed and synthesized the findings to construct the outline of this review.

## 3. Metabolic Evolution of Critically Ill Patients

Critical illness initiates a cascade of metabolic changes that evolve through the early acute catabolic phase and the subacute recovery phase. During the early acute phase, the body exhibits a hypermetabolic state, generally characterized by increased energy expenditure, insulin resistance, proteolysis, and lipolysis [18]. Systemic inflammation, induced or accompanied by elevated levels of stress hormones such as cortisol, catecholamines, and inflammatory cytokines, including tumor necrosis factor-alpha and interleukin-6, is the main driving force of metabolic alterations [19]. Catabolic reactions facilitate the mobilization of energy substrates to vital organs for survival but can lead to significant muscle wasting and nutrient depletion. Prolonged inflammation and oxidative stress lead to persistent tissue breakdown and impaired anabolic processes, while immobilization and disrupted energy and nutrient metabolism may augment muscle protein loss [20,21]. Sustained protein catabolism contributes to a negative nitrogen balance and further loss of lean body mass and various complications, a condition known as persistent inflammation–immunosuppression catabolism syndrome (PIICS) [1]. During this phase, gut barrier function often becomes compromised, increasing the risk of bacterial translocation and secondary infections, which can exacerbate systemic inflammation [22].

In the subacute or recovery phase, the metabolic profile gradually shifts towards anabolism and tissue repair. This phase is characterized by improved insulin sensitivity, reduced inflammatory markers, and a gradual restoration of muscle mass and organ function [18]. However, chronic critical illness characterized by immunosuppression and persistent metabolic dysregulation, such as mitochondrial dysfunction and altered substrate utilization, can impede full recovery and contribute to long-term physical and cognitive impairments [1,23,24].

**Figure 1:**
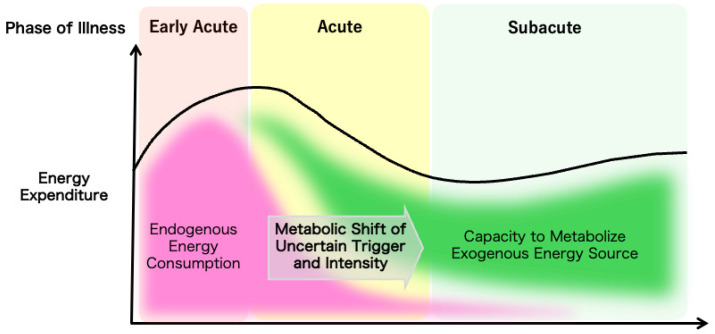
The ideal metabolic evolution in critical illness. The dynamic changes in the metabolic characteristics of critically ill patients are illustrated.

The overall evolution of a patient’s metabolic demands can be measured by indirect calorimetry as the total energy expenditure to guide nutrition therapy, but the trigger for the metabolic shift from catabolic to anabolic reactions and the mechanism for the regulation of the speed or the intensity of the shift are yet to be elucidated (Figure 1). Understanding the temporal progression of these metabolic changes is crucial for optimizing nutrition therapy. Tailoring nutritional interventions to the specific metabolic demands of each phase can enhance patient outcomes by mitigating muscle wasting, supporting immune function, and promoting recovery.

## 4. Gut Microbiota and Barrier Functions in the Critically Ill

To highlight the significance of nutrition therapy in critical illness, this section will first outline the fundamental mechanisms by which the gut maintains homeostasis through the interplay of microbiota and barrier functions.

### 4.1. Gut Microbiota

**Figure 2:**
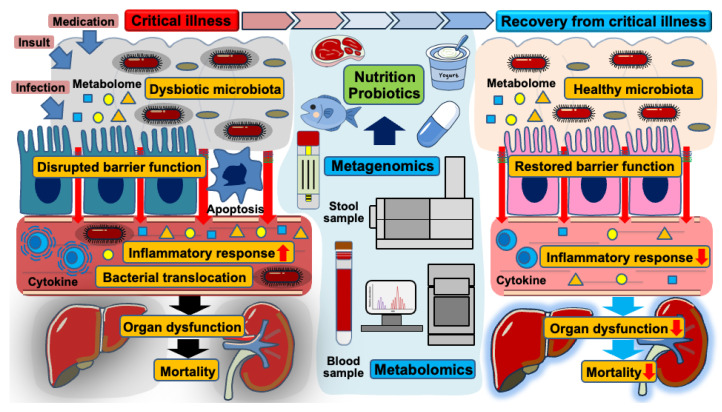
The role of gut microbiota, intestinal barrier dysfunction, and nutritional interventions in the transition from critical illness to recovery. This schematic illustration demonstrates the dynamic changes in gut microbiota and intestinal barrier function during critical illness and the impact of nutritional and probiotic interventions on recovery.

Trillions of gut microbiota inhabit human hosts, engaging in complex interactions that notably impact health and disease [25,26]. While intestinal microorganisms play essential roles in digesting nutrients and metabolizing various substances, they can also propagate inflammation when the microbial community shifts toward a pathobiome—a virulent state derived from a previously beneficial microbiota [27,28]. Advancements in metagenomic analysis through next-generation sequencing technology have deepened our understanding of the gut’s microbial composition [29]. Several factors such as stress, infections, and medication (e.g., antibiotics, analgesics, and anti-ulcer drugs) disrupt microbial balance, leading to intestinal dysbiosis [30]. This condition is characterized by a decline in protective bacterial populations, decreased microbial diversity, and an overgrowth of pathogenic microbiota [31,32]. In critical illness, overwhelming insults, infections, and various medications likely contribute to a dysbiotic state in the gut microbiota [11]. Such dysbiosis compromises the integrity of the intestinal barrier, facilitating bacterial translocation, and amplifying inflammatory responses, ultimately contributing to the progression of organ dysfunction [8,33]. Along with a loss of microbial diversity, virulent strains persist within the gut microenvironment, increasing the risk of mortality and nosocomial infections in critically ill patients (Figure 2) [34,35,36]. Previous observational studies using stool samples from patients with sepsis have reported a significant reduction in alpha-diversity, indicating a decreased abundance and diversity of microbial species following the onset of sepsis [6,37,38]. Beyond sepsis, other critical conditions such as trauma, out-of-hospital cardiac arrest, and burn injury similarly induce dysbiotic changes in the gut microbiota, further contributing to host vulnerability [39,40]. These findings suggest a strong association between gut dysbiosis and increased mortality in critically ill patients.

### 4.2. Gut Epithelium

The gut epithelium, consisting of a single layer of epithelial cells, serves as a critical barrier against invasive microorganisms while facilitating nutrient absorption. Beyond the epithelial cells, goblet cells, Paneth cells, intraepithelial lymphocytes, and mesenchymal cells harmoniously maintain intestinal homeostasis [41]. In critical illness, excessive inflammatory responses compromise gut barrier integrity, leading to increased permeability. A preclinical study using an abdominal sepsis model demonstrated an increased gut permeability during the early phase of systemic inflammation [42]. Similarly, clinical studies have shown elevated absorption rates for lactulose or multiple saccharides in critically ill patients, suggesting disrupted gut barrier function [43,44]. This increased gut permeability is largely attributed to alterations in the expression of tight junction (TJ) proteins, which play a crucial role in maintaining intestinal integrity. While the claudin family facilitates the transport of small molecules through pore pathway, zonula occludens-1, occludin, and junctional adhesion molecule A are involved in the passage of large molecules, including antigens and lipopolysaccharides, via leak pathways. The myosin light-chain kinase pathway modulates gut permeability by regulating actin–myosin contractions, thereby affecting TJ protein function [41,45]. In a murine model following cecal ligation and puncture surgery, sepsis induction led to increased claudin-2 expression and decreased occludin expression [22,42]. Within functional analyses, the deletion of claudin-2 reduced gut permeability and attenuated intestinal inflammation, whereas an occludin blockade exacerbated gut hyperpermeability with increased bacteremia during sepsis. These findings highlight the critical role of TJ protein dysregulation in gut barrier dysfunction in critical illness [22,46,47].

Gut hyperpermeability, in theory, increases the risk of bacterial translocation across a compromised mucosal barrier. Bacteria are thought to pass through an unrestricted pathway, regardless of size limitations [45,48]. However, clinical research examining portal vein blood samples from trauma patients has failed to provide direct evidence of bacterial translocation, as no significant bacterial presence was detected [49]. Despite the lack of direct proof, gut hyperpermeability remains a central hallmark in the pathophysiology of critical illness [8]. Even without detectable bacterial translocation, bacteria and their components can still trigger systemic inflammation. Supporting this concept, bacteria activate immune cells in a hyperinflammatory environment, amplifying inflammatory responses, and contributing to the progression of organ dysfunction [46,50,51,52].

### 4.3. Gut Immune System

Beneath the epithelial surface lies a complex immune network comprising lamina propria immune cells, Peyer’s patches, and mesenteric lymph nodes. These structures house lymphocytes, macrophages, and dendritic cells that play a pivotal role in immune defense [53,54]. The gut immune system also possesses immunological memory shaped by microbial exposure [55]. This is highlighted by the fact that individuals lacking a properly developed gut microbiota fail to establish a robust immune system, rendering them more susceptible to infections [56,57]. Therefore, the gut microbiome is not only essential for digestion and metabolism but also plays a fundamental role in the development of the immune system and the maintenance of host defense.

## 5. Nutrition as a Modulator of Gut-Derived Metabolism

To mitigate the effects of illness on the gut microenvironment, various strategies are being explored in critical care settings, among which nutritional interventions show particular promise for improving patient outcomes [11,58].

### 5.1. Nutrition and Gut Microbiota

Under normal physiological conditions, dietary composition profoundly shapes the gut microbiome [59]. Carbohydrate-rich diets tend to enrich *Prevotella* populations, while protein- and animal fat-rich diets typically promote the growth of Bacteroides [60]. Specifically, high-fat diets alter the microbial balance, resulting in a decreased proportion of *Bacteroidetes* and increased proportions of *Firmicutes* and *Proteobacteria* [61]. In addition, high-fat diets contribute to gut hyperpermeability by disrupting TJ proteins and exacerbating intestinal inflammation [62,63,64]. Notably, these microbiome alterations may be reversible, as demonstrated by studies indicating that dietary modifications can restore the microbiome’s composition to its baseline state [65]. Dietary fiber, an indigestible carbohydrate derived from plants, increases the abundance of *Lactobacillus* and *Bifidobacterium* [66], whereas low-fiber diets are associated with a reduced proportion of *Bifidobacterium* [67]. Moreover, low-fiber diets diminish the mucus layer, leading to increased gut permeability, enhanced local inflammation, and intestinal dysbiosis [68,69,70].

In critical care settings, early enteral nutrition is often recommended to support gut integrity and reduce the risk of infectious complications through prevention [71]. Immunonutrition formulas supplemented with glutamine, arginine, omega-3 fatty acids, or nucleotides have shown potential benefits in modulating systemic inflammation and improving clinical outcomes [72,73]; however, these interventions must be carefully tailored to each patient’s condition, as the benefits can vary depending on the severity of illness and underlying comorbidities [74]. Moreover, modulation of the gut microbiome through the administration of prebiotics, probiotics, or synbiotics may offer additional benefits for critically ill patients. Prebiotics, non-digestible fibers or compounds that serve as food for beneficial gut bacteria, can enhance the production of beneficial metabolites such as SCFAs by selectively stimulating the growth of commensal bacteria [75]. In murine models of sepsis, prebiotic supplementation improved survival by modulating the gut microbiome and reducing systemic inflammation; notably, this survival benefit was microbiota-dependent, as it was abolished by antibiotic treatment [76]. Although a randomized controlled trial in critically ill patients failed to demonstrate an increase in beneficial microbiota following supplementation with oligofructose/inulin [77], clinical studies have suggested that a fiber-rich diet may promote a favorable microbial composition and help preserve the mucosal barrier during critical illness [78,79]. Probiotics, live beneficial bacteria or yeasts, including *Lactobacillus* and *Bifidobacterium* strains, may help stabilize the gut microbiota and support immune function [80]. In preclinical models of sepsis, probiotic administration has been shown to alleviate gut barrier dysfunction and intestinal inflammation, leading to improved survival outcomes [81,82,83]. Furthermore, a systematic review of randomized controlled trials in critically ill patients reported that probiotics were effective in preventing diarrhea and infectious complications, although no significant impact on mortality was observed [84,85,86]. While probiotics have potential in improving gut microenvironment and reducing infection rates, clinicians should weigh possible risks, including probiotic-related translocation or sepsis in critically immunocompromised patients [87,88]. Synbiotics, which combine prebiotics and probiotics, are designed to exert synergistic effects on gut health [89]. From a microbiota perspective, synbiotic supplementation has been associated with an increased proportion of fecal *Bifidobacteria*. Additionally, a previous study demonstrated that synbiotics reduced the incidence of enteritis and ventilator-associated pneumonia in patients with sepsis through the modulation of gut microbiota (Table 1) [90]. To accelerate clinical applications, these promising findings warrant validation in large-scale clinical trials.

### 5.2. Gut-Derived Metabolites

The gut microbiome produces a diverse array of metabolites, including SCFAs, bile acids, trimethylamine N-oxide (TMAO), and tryptophan-derived compounds, which play pivotal roles in regulating intestinal inflammation and maintaining mucosal homeostasis by modulating immune cell function and epithelial barrier integrity [91,92,93]. SCFAs, such as acetate, propionate, and butyrate, not only serve as an energy source for gut epithelial cells but also promote the expansion of regulatory T cells and suppress pro-inflammatory cytokine production [94,95]. Bile acids, in turn, are transformed from primary to secondary forms by gut microbes, subsequently acting on nuclear receptors to modulate metabolic and immune pathways [96]. Additionally, gut bacteria convert dietary components such as phosphatidylcholine and carnitine into trimethylamine, which is then oxidized in the liver to produce TMAO, a metabolite associated with atherosclerosis and other cardiovascular diseases [97]. Furthermore, tryptophan metabolites regulate serotonin production, modulate immune responses, and preserve intestinal barrier integrity, thereby affecting the gut–brain axis [98,99,100].

Metabolomics have provided valuable insights into the impact of dysbiosis on diseases by comprehensively profiling small molecules. In patients with inflammatory bowel disease, decreased fecal concentrations of SCFAs have been observed, potentially exacerbating disease severity [101,102]. Similarly, patients with sepsis had significantly lower stool SCFA concentrations (propionic, acetic, butyric, and isobutyric acids) than controls. This study suggests that lower SCFA levels may impact gut integrity and barrier function in critical illness [103]. Emerging evidence suggests that these microbiota-derived metabolites are absorbed in the intestines and enter systemic circulation, contributing to extraintestinal diseases including atherosclerosis, kidney disease, and psychiatric disorders [104,105]. In addition, fluctuations in blood metabolites appear to reflect changes in gut microbiota, implicating the dynamic crosstalk between circulating metabolites and microbial activity in the gut [106,107]. In critically ill patients, a previous study using stool samples revealed an association between tryptophan metabolism, gut dysbiosis, and sepsis severity, highlighting the critical role of gut-derived metabolites in sepsis pathophysiology [6]. Another study examined the relationship between TMAO, a metabolite associated with an increased risk of cardiovascular diseases, and mortality and nutritional state in patients with sepsis. Intriguingly, an inverse correlation between plasma TMAO concentrations and the severity of sepsis and malnutrition was observed, suggesting that the functional roles of gut-derived metabolites may vary across different diseases [108].

While functional analyses of these metabolites remain challenging in clinical studies, preclinical research has demonstrated the critical roles of gut-derived metabolites in critical illness. In a murine sepsis model, sodium butyrate reduced the gene expression of high-mobility group box 1, decreased organ damage markers, and improved survival rates, suggesting that sodium butyrate may protect against multi-organ dysfunction in sepsis [109]. Another study investigating the administration of SCFAs, including acetate, propionate, and butyrate, demonstrated improved cognitive function and reduced neuroinflammation [110]. Furthermore, indole-3-propionic acid, a microbial metabolite, showed a benefit to survival during sepsis by reducing inflammation and modulating gut microbiota composition, increasing *Bifidobacteriaceae* and reducing *Enterobacteriaceae* [111]. These findings suggest that gut-derived metabolites, or postbiotics, may serve as therapeutic alternatives to probiotics or fecal microbiota transplantation to improve gut dysbiosis and clinical outcomes in critically ill patients (Table 1) [13,112]. Although the direct relationship between gut-derived metabolites and the progression of organ dysfunction remains to be determined, these metabolic signatures hold potential for enhancing diagnostics, prognostics, and personalized treatment strategies in critical illness.

**Table 1:** Microbiota- and metabolome-based nutritional interventions in critically ill patients.

Interventions	Description	Findings	Limitations	Reference
Prebiotics	Prebiotics stimulate beneficial bacterial growth and production of short-chain fatty acids (SCFAs).	Improved gut barrier and reduced inflammation in small trials	Lack of large-scale studies	[76,77]
Probiotics	Probiotics possibly restore gut microbiota balance, enhance intestinal barrier function, and reduce intestinal inflammation.	Mixed results in RCTs for prevention of infections and reduction in mortality	Strain-specific effects Risk of bacterial translocation in immunocompromised patients	[80,81,82,83,84,85,86,87,88]
Synbiotics	Synbiotics, combined agents of prebiotics and probiotics, enhance colonization and SCFA production.	Reduction in ventilator-associated pneumonia and enteritis in some studies	Complexity in formulation and dosing Variability in patient response	[89,90]
Postbiotics	Direct delivery of bacterial metabolites (e.g., SCFAs) modulates immune response and gut integrity.	Reduction in inflammation in animal and small human studies	Optimal dosage and delivery method not established Lack of clinical studies	[109,110,111,112]
Fiber-Enriched Formulas	Fiber-enriched formulas promote fermentation by gut microbiota to produce SCFAs and enhance gut integrity.	Reduction in diarrhea and improved gut function	Tolerance issues Lack of standardized formulations	[78,79]

### 5.3. Nutritional Metabolomics

To obtain a better understanding of the complex interplay between nutrition, gut microbiota, and the metabolome, multi-omics approaches, integrating metagenomics, transcriptomics, proteomics, and metabolomics, have emerged as powerful tools. These approaches provide a comprehensive interpretation of microbiota-derived metabolites and their impact on health and diseases [15,16,17]. Building on these discoveries, various nutritional approaches have been investigated for their potential to modulate gut-derived metabolism and restore a healthy microbial balance [113,114]. While the ultimate goal is to develop optimal nutritional interventions, current advancements allow physicians to visualize metabolomic profiles, enabling personalized dietary and therapeutic strategies to improve outcomes in critically ill patients [12,14]. A previous study investigated the impact of individualized versus standard energy supply on serum metabolomics in critically ill patients receiving enteral or parenteral nutrition. Individualized energy supply based on indirect calorimetry altered metabolite profiles, reducing markers of catabolism and suggesting that metabolomics could help identify metabolic phases in critical illness [113]. Through these innovations, nutrition offers substantial potential to regulate gut-derived metabolism, enhance patient outcomes, and drive advancement in the critical care field.

## 6. Future Expectations for Nutrition from the Metabolic Point of View

Future nutrition therapy may focus not only on supplementing the nutritional needs of critically ill patients but rather they can take a step further towards metabolic modulation strategies to ameliorate the metabolic burden of the critical illness and mitigate catabolic reactions for improved clinical and functional recovery. A modest approach may be through the use of targeted prebiotics, probiotics, or synbiotics to restore microbial balance and enhance gut barrier integrity [115]. Leveraging insights from extensive gut microbiota research and next generation metabolomics and metagenomics studies may open the door to innovative metabolic modulation strategies.

While metabolomics may offer promising pathways for personalizing nutrition therapy [12], comprehensive profiling of the metabolic structures of critically ill patients may enable clinicians to identify specific nutrient deficiencies, metabolic derangements, and biomarkers to predict disease progression or recovery [116]. Such a precise approach can also facilitate tailored nutritional interventions to address individual metabolic needs, optimize energy utilization, and attenuate catabolic reactions to improve clinical and functional outcomes.

Metabolic modulation targeting key metabolic pathways to enhance the resilience and recovery of critically ill patients represents another frontier in nutrition therapy. Tentative strategies may involve the use of specialized nutrients such as omega-3 fatty acids, glutamine, and arginine to modulate inflammatory responses, support mitochondrial function, and promote tissue repair [117,118,119]. Alternatively, simple protocols to optimize energy and protein provision have also been explored for their potential to induce beneficial metabolic adaptations, i.e., mitigating catabolism, thus actuating anabolic response, improving insulin sensitivity, and sustaining autophagy [119,120].

Integrating these emerging approaches into clinical practice will require robust research to elucidate the mechanisms underlying their effects and to establish evidence-based strategies. A deeper understanding of the interplay between nutrition, metabolism, and critical illness will pave the way for innovative therapies to improve not only the survival of but also the subsequent quality of life of critically ill patients.

## 7. Limitations

This review has several limitations. First, although we aimed to include a comprehensive range of studies, there may have been selection bias due to the exclusion of conference abstracts and non-peer-reviewed sources. Second, the review focused on metabolic and gut microbiota-related outcomes in critically ill patients, potentially overlooking other important clinical factors. Lastly, the heterogeneity of the included studies in terms of patient populations, nutritional interventions, and outcome measures may limit the generalizability of the findings.

## 8. Conclusions

Critical illness induces profound metabolic alterations, and the interplay between systemic inflammation, gut microbiota dysbiosis, and impaired gut barrier function further exacerbates these metabolic challenges, increasing the risk of organ dysfunction and prolonging recovery. Nutritional therapy, particularly early enteral nutrition, plays a pivotal role in modulating these metabolic responses by preserving gut integrity, mitigating catabolism, and supporting immune function. Emerging evidence highlights the potential of targeted nutritional strategies, including prebiotics, probiotics, and microbiota-derived metabolites, to restore microbial balance and optimize host metabolism. Advances in multi-omics technologies offer new insights into personalized nutrition approaches tailored to individual metabolic needs. Future research should focus on refining these strategies to enhance recovery, reduce complications, and improve long-term functional outcomes in critically ill patients. By integrating precision nutrition with an improved understanding of gut-derived metabolism, critical care nutrition may evolve beyond conventional caloric support into a key therapeutic intervention for metabolic modulation and enhanced patient survival.
